# The Indoleamine-2,3-Dioxygenase (IDO) Inhibitor 1-Methyl-D-tryptophan Upregulates IDO1 in Human Cancer Cells

**DOI:** 10.1371/journal.pone.0019823

**Published:** 2011-05-20

**Authors:** Christiane A. Opitz, Ulrike M. Litzenburger, Uta Opitz, Felix Sahm, Katharina Ochs, Christian Lutz, Wolfgang Wick, Michael Platten

**Affiliations:** 1 Department of Neurooncology, Neurology Clinic and National Center for Tumor Diseases, University Hospital of Heidelberg, Heidelberg, Germany; 2 Experimental Neuroimmunology Unit, German Cancer Research Center, Heidelberg, Germany; 3 Heidelberg Pharma AG, Ladenburg, Germany; 4 Department of Neuropathology, Institute of Pathology, University Hospital of Heidelberg and Clinical Cooperation Unit Neuropathology, German Cancer Research Center, Heidelberg, Germany; 5 Clinical Cooperation Unit Neurooncology, German Cancer Research Center, Heidelberg, Germany; Governmental Technical Research Centre of Finland, Finland

## Abstract

1-methyl-D-tryptophan (1-D-MT) is currently being used in clinical trials in patients with relapsed or refractory solid tumors with the aim of inhibiting indoleamine-2,3-dioxygenase (IDO)-mediated tumor immune escape. IDO is expressed in tumors and tumor-draining lymph nodes and degrades tryptophan (trp) to create an immunsuppressive micromilieu both by depleting trp and by accumulating immunosuppressive metabolites of the kynurenine (kyn) pathway. Here we show that proliferation of alloreactive T-cells cocultured with IDO1-positive human cancer cells paradoxically was inhibited by 1-D-MT. Surprisingly incubation with 1-D-MT increased kyn production of human cancer cells. Cell-free assays revealed that 1-D-MT did not alter IDO1 enzymatic activity. Instead, 1-D-MT induced IDO1 mRNA and protein expression through pathways involving p38 MAPK and JNK signalling. Treatment of cancer patients with 1-D-MT has transcriptional effects that may promote rather than suppress anti-tumor immune escape by increasing IDO1 in the cancer cells. These off-target effects should be carefully analyzed in the ongoing clinical trials with 1-D-MT.

## Introduction

In recent years tryptophan (trp) degradation has received increasing attention as a potent immunosuppressive mechanism involved in the maintenance of immunological tolerance. The trp-degrading enzyme indoleamine-2,3-dioxygenase (IDO) has been implicated in maternal tolerance towards allogeneic concepti [1], controlling autoimmune diseases [2], [3] and chronic infection [4], as well as promoting tumor immune escape [5], [6], [7]. IDO-mediated trp degradation is not restricted to tumor cells [7] but is also detected in tumor-draining lymph nodes [8]. In both tumor-draining lymph nodes and tumors, IDO1 creates local tolerance by directly suppressing T-cell responses and enhancing immunosuppression mediated by regulatory T cells (T*reg*) [6]. IDO is chronically activated in many cancer patients [9] and its expression or enzyme activity correlates with a poor prognosis in patients with various cancers such as ovarian carcinoma [10], [11], endometrial carcinoma [12], [13], hepatocellular carcinoma [14] and colorectal carcinoma [15].

Despite the bulk of evidence supporting a role for IDO in promoting tumor formation and tumor immune escape, there have been studies showing an anti-tumor activity of IDO1. The induction of IDO1 has been described as a mechanism by which Interferon (IFN)-γ inhibits proliferation of malignant cells [16], [17]. Some animal experiments demonstrated that IDO1 expression was positively associated with the elimination of malignant cells [18], [19]. These findings were corroborated in clinical studies showing that despite being a strong inducer of IDO1, IFN-γ was effective in the therapy of ovarian carcinoma and bladder cancer [20], [21], [22]. In addition, IDO1 expression in hepatocellular carcinoma specimens and in endothelial cells of renal cell carcinoma positively correlated with progression-free survival and long-term survival, respectively [23], [24]. There thus remains uncertainty about the clinical relevance of IDO1 expression in tumors.

In preclinical studies the IDO-inhibitor 1-methyl-tryptophan (1-MT) reduced the tumor volume of mice preimmunized with a tumor antigen [7] and - in combination with chemotherapeutic agents - caused regression of established murine breast cancers [5]. Inhibition of IDO in combination with chemotherapy or as a vaccine adjuvant therefore represents an attractive approach for cancer immunotherapy [5], [6], [7], [25], [26]. Recently a novel IDO isoform, termed IDO2 was discovered, which - like IDO1 - is expressed in tumors and tumor-draining lymph nodes [27]. The third trp-degrading enzyme in humans, tryptophan-2,3-dioxygenase (TDO) is mainly expressed in the liver and regulates trp concentrations after nutritional trp uptake. The IDO inhibitor 1-MT exists as two stereoisomers, 1-D-MT and 1-L-MT. Most preclinical studies have employed the racemic mixture 1-D/L-MT to inhibit IDO. Recent studies have shown that IDO1 is the preferential target of 1-L-MT, while 1-D-MT preferentially inhibits IDO2 [26], [27], [28], [29]. 1-D-MT is currently used in phase I clinical trials as an adjunct to conventional chemotherapy based on preclinical studies in mouse models of cancer. We were interested in the immunomodulatory effects of 1-D-MT in IDO1-positive human cancer cells.

## Results

### 1-D-MT induces immunosuppression of human cancer cells

**Figure 1:**
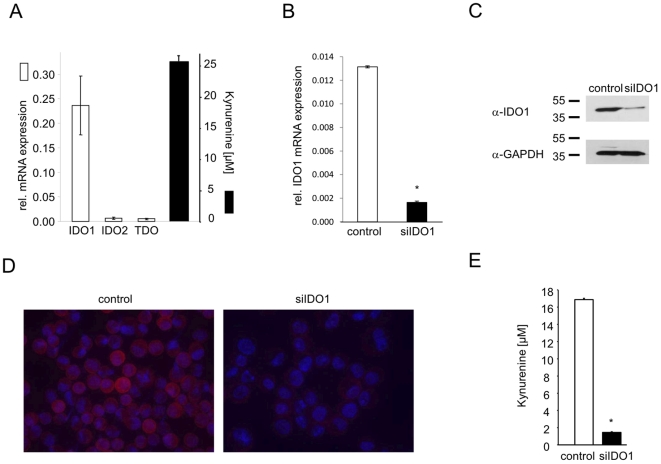
SKOV-3 ovarian carcinoma cells constitutively degrade trp via indoleamine-2,3-dioxygenase-1 (IDO1). (**A**) Relative mRNA expression of the three trp-degrading enzymes IDO1, IDO2 and tryptophan-2,3-dioxygenase (TDO) (white bars) and kyn production (black bar) of SKOV-3 cells, measured by quantitative RT-PCR and high performance liquid chromatography (HPLC). (**B**) Knockdown of *IDO1* mRNA by siRNA measured by qRT-PCR. (**C**) Western blot analysis showing IDO1 protein expression in SKOV-3 cells with siRNA mediated knockdown of IDO1 in comparison to controls. (**D**) Immunocytochemistry (red, IDO1 staining; blue, DAPI nuclear staining) of control SKOV-3 cells and SKOV-3 cells with IDO1 knockdown. (**E**) Kyn release of SKOV-3 cells after knockdown of IDO1 in comparison to controls. Experiments were performed at least in triplicate. Data are mean ± SEM. * (p<0.05).

**Figure 2:**
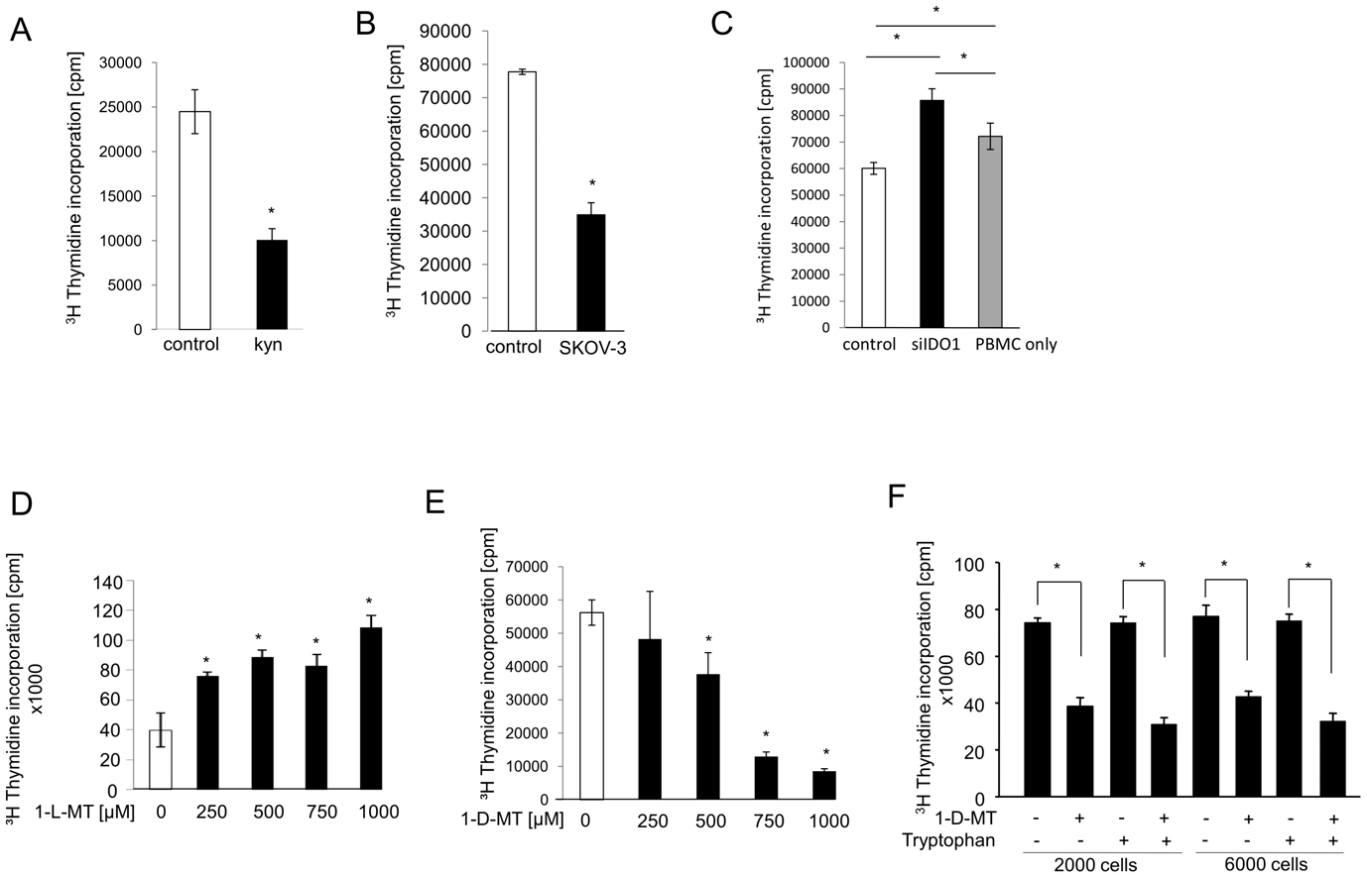
1-D-MT reduces T cell proliferation in cocultures of SKOV-3 cells with mixed leukocyte reactions. (**A**) Alloreactive T cell proliferation after addition of 25 µM kyn to mixed leukocyte reactions (MLR). (**B**) Alloreactive T cell proliferation in the presence of 6000 SKOV-3 cells. (**C**) T cell proliferation in MLR cocultured with 2000 control SKOV-3 cells (white bar) or 2000 SKOV-3 cells with a knockdown of IDO1 (black bar). (**D**) T cell proliferation in cocultures of MLR with 2000 SKOV-3 cells after addition of increasing concentrations of 1-L-MT. (**E**) T cell proliferation in cocultures of MLR with 2000 SKOV-3 cells after addition of increasing concentrations of 1-D-MT. (**F**) Representative result of MLR/SKOV-3 coculture experiments with PBMC from five different donors and 2000 or 6000 SKOV-3 cells. Cells were treated with or without 1 mM 1-D-MT in combination with or without 250 µM trp. Proliferation was measured by ^3^[H] methylthymidine uptake. Experiments were performed at least in triplicate. Data are mean ± SEM. * (p<0.05).

**Figure 3:**
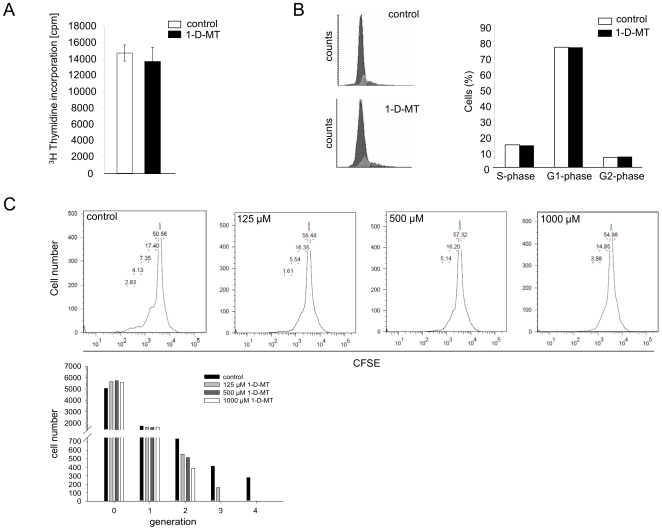
1-D-MT does not inhibit the proliferation or cell cycle progression of SKOV-3 cells. (**A**) ^3^[H] methylthymidine incorporation of SKOV-3 cells treated with 1 mM 1-D-MT (black bar) or vehicle (white bar) for 6 days. (**B**) Cell cycle analysis of SKOV-3 cells treated with 1 mM 1-D-MT or vehicle for 48 h. (**C**) Proliferation analysis of CFSE-stained lymphocytes from 6 day cocultures of MLR with 2000 SKOV-3 cells, treated with indicated concentrations of 1-D-MT (upper panel). Plot of the cell numbers in each generation of the above experiment (lower panel).

SKOV-3 cells constitutively degrade trp to kyn and express high levels of IDO1 mRNA while IDO2 and TDO mRNA are expressed at low levels (Fig. 1A). Knockdown of IDO1 by siRNA in SKOV-3 cells decreased IDO1 mRNA expression by 87.5% (Fig. 1B) leading to a strong reduction in IDO1 protein expression as evidenced by Western Blot (Fig. 1C) and immunocytochemistry (Fig. 1D). Finally, kyn production was inhibited by 91.4% in the IDO1 knockdown cells in comparison to the mean kyn production of SKOV-3 cells transfected without siRNA or with a non-targeting siRNA control (Fig. 1E), suggesting that IDO1 is mainly responsible for the constitutive kyn production in SKOV-3 cells. To determine the effect of 1-MT treatment on the immunomodulatory phenotype of cancer cells, SKOV-3/MLR coculture experiments were performed. Addition of kyn (Fig. 2A) or the presence of SKOV-3 cells (Fig. 2B) inhibited alloreactive T cell proliferation in MLR. Knockdown of IDO1 by siRNA not only reversed the SKOV-3 cell mediated suppression of T cell proliferation, but even increased T cell proliferation (Fig. 2C), probably due to additional allogeneic stimulation of the T cells by the IDO-deficient SKOV-3 cells. Next we tested the effects of the two stereoisomers of 1-MT. Addition of 1-methyl-L-tryptophan (1-L-MT) also reversed the suppression of T cell proliferation in the SKOV-3/MLR coculture experiments (Fig. 2D). Surprisingly, T cell proliferation was not enhanced but inhibited in cocultures treated with 1-methyl-D-tryptophan (1-D-MT, Fig. 2E). Addition of trp did not alter this inhibition, indicating that trp depletion is not involved in this paradoxical effect of 1-D-MT (Fig. 2F). Next we analyzed the effect of 1-D-MT on the cell cycle progression and proliferation of SKOV-3 cells, as an inhibitory effect of 1-D-MT on SKOV-3 cells could explain the reduced ^3^H thymidine uptake in the coculture experiments (Fig. 3). However, 1-D-MT altered neither ^3^H thymidine uptake (Fig. 3A) nor cell cycle progression of SKOV-3 cells (Fig. 3B). To rule out, that 1-D-MT might have inhibited ^3^H thymidine uptake of SKOV-3 cells only when these were cultured in the MLR, T cell proliferation in cocultures of SKOV-3 cells with MLR in the presence of different concentrations of 1-D-MT was measured by CFSE staining and flow cytometry (Fig. 3C). 1-D-MT concentration-dependently inhibited T cell proliferation also in these assays (Fig. 3C), thus confirming that 1-D-MT inhibits T cell proliferation and not the proliferation of SKOV-3 cells in the cocultures.

### 1- D-MT increases kyn production in human cancer cells

**Figure 4:**
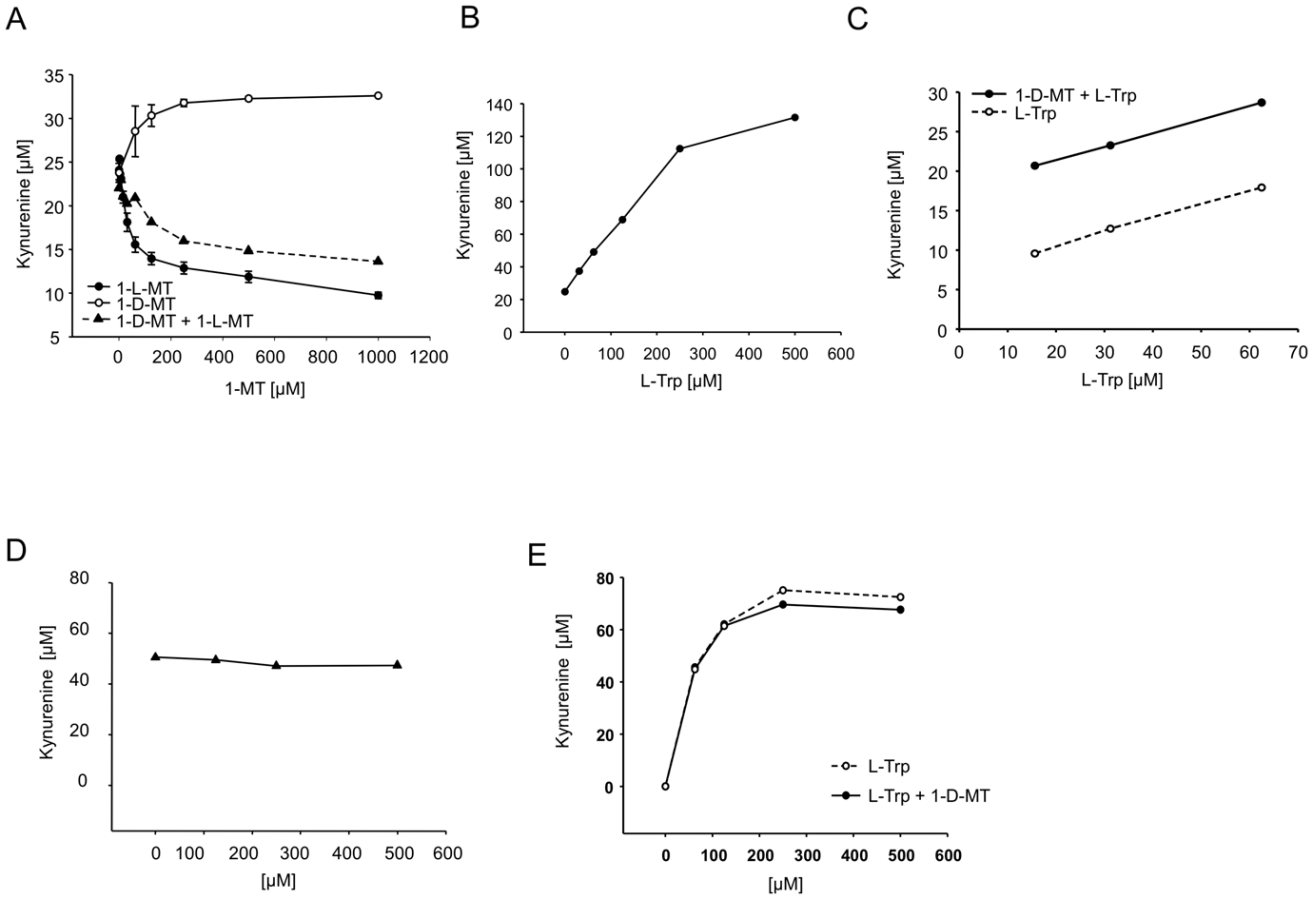
Increased kyn release of SKOV-3 cells upon 1-D-MT treatment. (**A**) Kyn concentrations released by SKOV-3 cells after treatment with 1-D-MT (white circles), 1-L-MT (black circles) and the racemic mixture of 1-MT (black triangles) measured after 48 h by HPLC. (**B**) Kyn release of SKOV-3 cells in response to 500 µM 1-D-MT in the presence of increasing trp concentrations. (**C**) Kyn release of SKOV-3 cells treated with different concentrations of trp alone (open circles) or in combination with 1 mM 1-D-MT (filled circles) measured after 48 h by HPLC. (**D**) Kyn production in IDO1 enzymatic assays performed in the presence of 100 µM trp in combination with increasing 1-D-MT concentrations. (**E**) Kyn production of IDO1 enzyme in the presence of increasing concentrations of trp alone (open circles) or in combination with 1-D-MT (filled circles). Experiments were performed in triplicate. Data are mean ± SEM. * (p<0.05).

We then tested the effect of 1-MT on the kyn production of SKOV-3 cells. Surprisingly, 1-D-MT concentration-dependently increased kyn formation (Fig. 4A), while its stereoisomer 1-L-MT inhibited kyn formation as expected (Fig. 4A). The racemic mixture of 1-MT, which has been used in many studies, including those that have established IDO1 as an immunosuppressive enzyme, inhibited kyn formation, albeit less than 1-L-MT alone (Fig. 4A). As trp concentrations in the media may have limited the increase in kyn production, we also measured the kyn concentrations produced by SKOV-3 cells in response to 1-D-MT in the presence of increasing trp concentrations. Under these conditions much higher kyn concentrations were reached (Fig. 4B), suggesting that the plateau observed above concentrations of about 250 µM 1-D-MT (Fig. 4A) was due to limited trp availability. Trp concentrations in cell culture media usually vary between 12 and 20 µM while concentrations in human serum range between 50 and 70 µM. Kyn formation in cells treated with 1-D-MT was more pronounced when trp concentrations present in human serum (62.5 µM) rather than trp concentrations present in the cell culture media (15 µM) were used (Fig. 4C). However, the fold increase in kyn by addition of trp was equal in cells treated with or without 1-D-MT (Fig. 4C). To further test whether 1-D-MT directly influences IDO1 enzymatic activity we measured IDO1-mediated kyn production in SKOV-3 cell extracts. 1-D-MT did not alter kyn formation regardless whether trp was present at a fixed concentration of 100 µM (Fig. 4D) or at concentrations equimolar to 1-D-MT (Fig. 4E), suggesting that the increase in kyn formation by 1-D-MT in SKOV-3 is not mediated by a direct effect of 1-D-MT on IDO1 enzymatic activity.

### IDO1 expression is enhanced by 1-D-MT in human cancer cells

**Figure 5:**
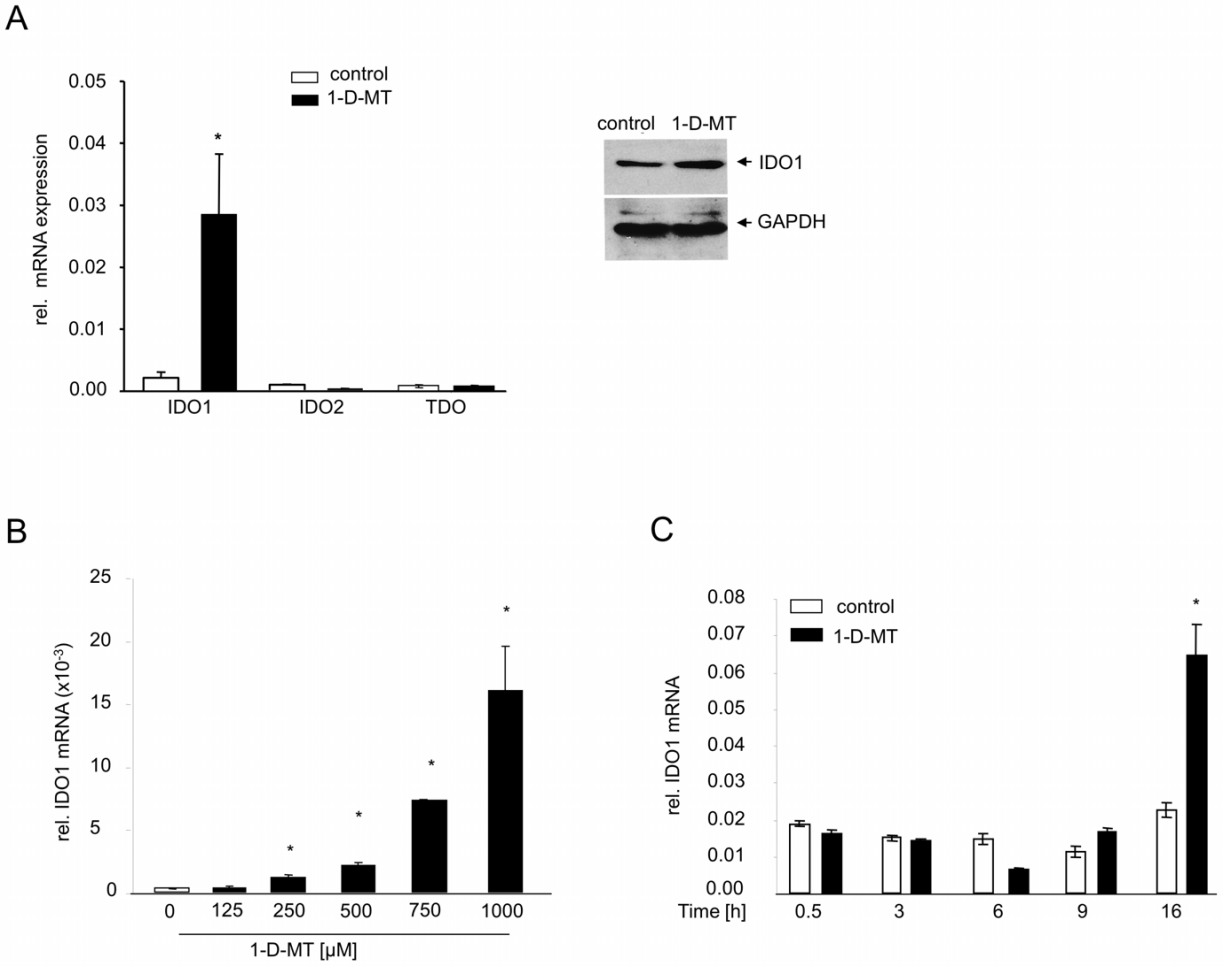
Upregulation of IDO1 mRNA by 1-D-MT in SKOV-3 cells. (**A**) Left panel: mRNA expression of IDO1, IDO2 and TDO in SKOV-3 cells after treatment with 1 mM 1-D-MT, analyzed after 24 h by qRT-PCR. Right panel: Western Blot analysis of IDO1 expression in SKOV-3 cells performed after 48 h 1 mM 1-D-MT. GAPDH served as loading control. (**B**) IDO1 mRNA expression in response to increasing concentrations of 1-D-MT measured after 24 h by qRT-PCR. (**C**) Time course analysis of IDO1 mRNA induction by 1 mM 1-D-MT, analyzed by qRT-PCR. Experiments were performed in triplicate. Data are mean ± SEM. * (p<0.05).

**Figure 6:**
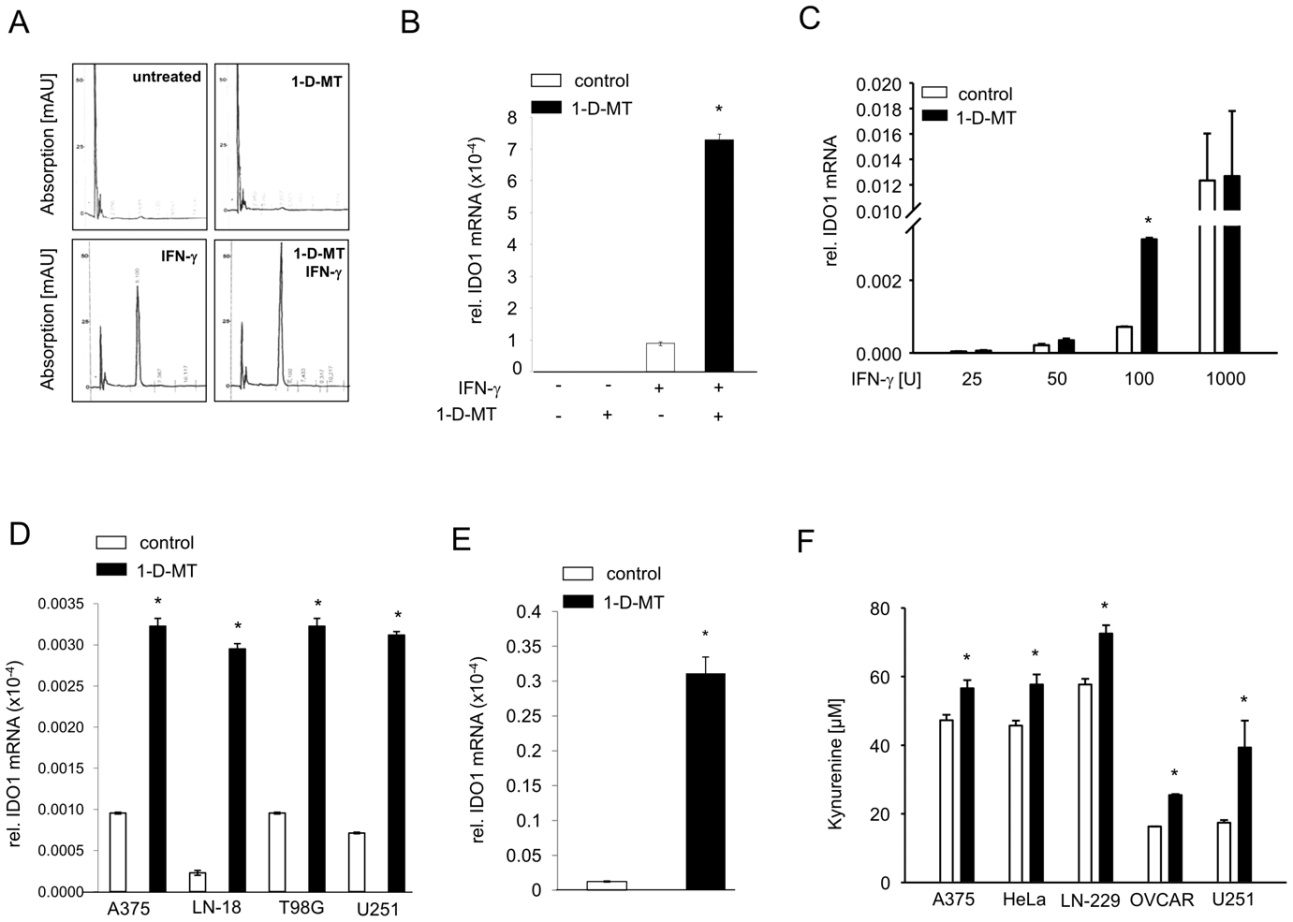
1-D-MT upregulates IDO1 expression and kyn release induced by different concentrations of IFN-γ in diverse cancer cells. (**A**) Representative HPLC graphs of kyn production of HeLa cells, which were either untreated, treated with 1 mM 1-D-MT and/or 1000 U IFN-γ for 72 h. Absorption of kyn was measured at 365 nm. (**B**) In untreated HeLa cells 1 mM 1-D-MT did not induce *de novo* IDO1 mRNA, but increased IDO1 mRNA after its induction by 1000 U IFN-γ mRNA expression was analyzed by qRT-PCR 24 h after treatment. (**C**) Representative example of the effect of different IFN-γ concentrations on IDO1 mRNA induction by 1-D-MT, shown in U251 glioma cells. (**D**) IDO1 mRNA expression of indicated cell lines that were stimulated for 24 h with appropriate concentrations of IFN-γ alone (white bars) or in combination with 1 mM 1-D-MT (black bars). (**E**) Representative example of IDO1 mRNA induction by 200 µM 1-D-MT in IFN-γ-stimulated T98G glioma cells. (**F**) Kyn release of indicated cell lines that were stimulated for 72 h with appropriate concentrations of IFN-γ alone (white bars) or in combination with 1 mM 1-D-MT (black bars), measured by HPLC. Experiments were performed in triplicate. Data are mean ± SEM. * (p<0.05).

Next, we investigated whether 1-D-MT influenced the expression of trp-metabolizing enzymes. To our surprise, we found that 1-D-MT increased IDO1 mRNA and protein in SKOV-3 cells, while IDO2 and TDO remained unaltered (Fig. 5A). Upregulation of IDO1 mRNA was concentration-dependent (Fig. 5B) and was first detected after 16 h of incubation with 1-D-MT (Fig. 5C). Importantly, the IDO1-promoting effects were not restricted to SKOV-3 cells. While 1-D-MT did not induce *de novo* IDO1 mRNA expression and kyn production in IDO1-negative HeLa cervical carcinoma cells, it increased IDO1 mRNA and kyn production after induction of IDO1 expression and kyn production by IFN-γ(Fig. 6A,B). Interestingly, the 1-D-MT-mediated upregulation of IDO1 mRNA in many IDO1-negative cancer cells was differentially dependent of the concentration of IFN-γ that was used to induce *de novo* expression of IDO1 (Fig. 6C). After stimulation with appropriate IFN-γ concentrations, 1-D-MT increased IDO1 mRNA and kyn production in a panel of different cancer cells (Fig. 6C–F), indicating a universal mechanism of 1-D-MT-mediated activation of IDO1.

### 1-D-MT-mediated IDO1 expression involves JNK and p38 MAPK

**Figure 7:**
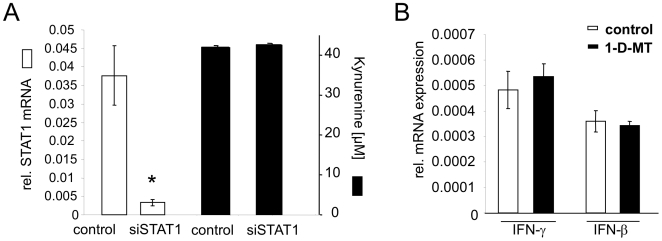
STAT1 signaling is not involved in the IDO1 upregulation in response to 1-D-MT. (**A**) Knockdown of STAT1 mRNA by si-RNA (white bars) did not affect kyn release (black bars) of 1-D-MT (1 mM) treated SKOV-3 cells. (**B**) Analysis of IFN-γ and IFN-β mRNA expression in SKOV-3 cells after stimulation with 1 mM 1-D-MT for 24 h.

**Figure 8:**
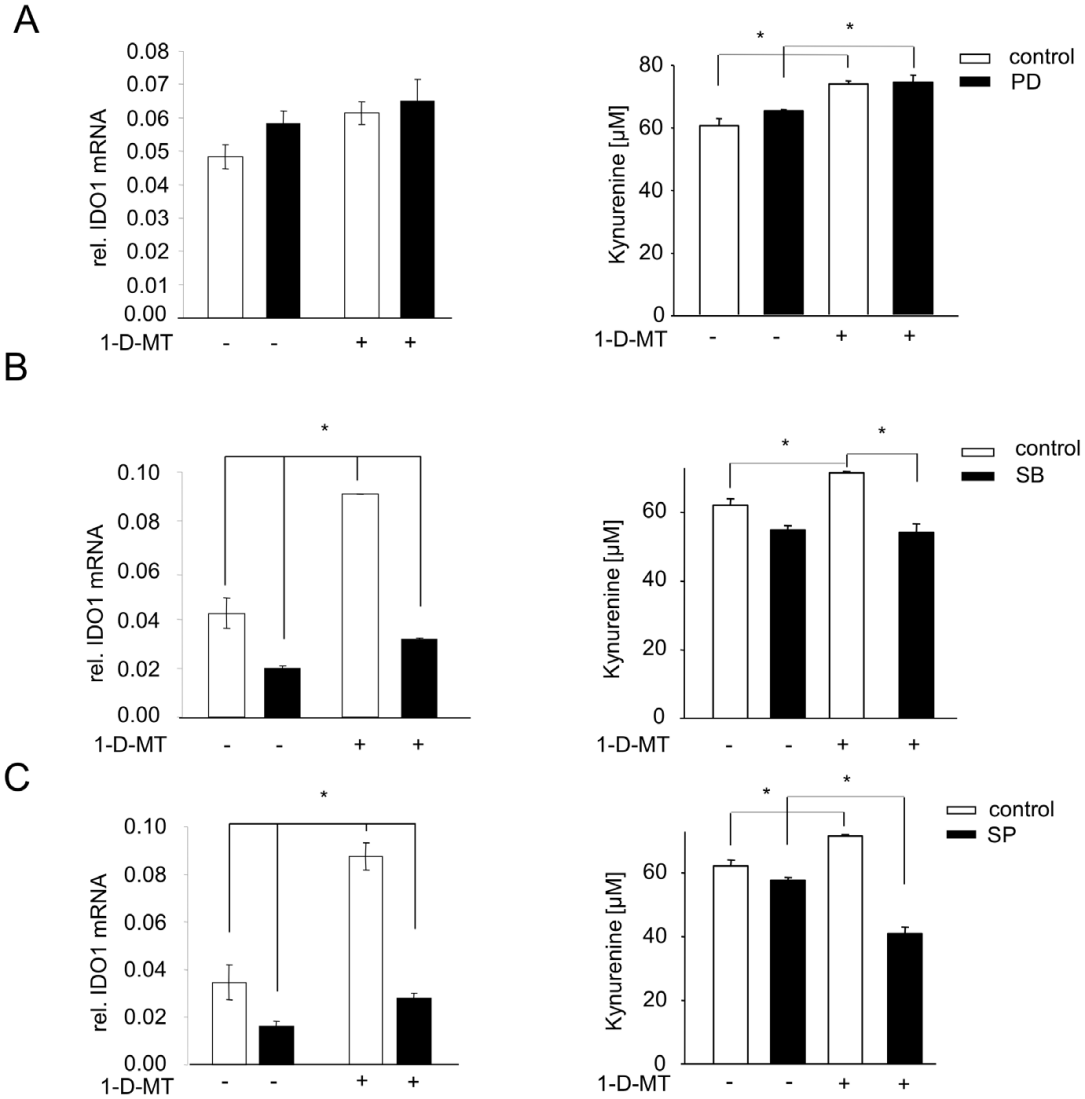
p38 mitogen-activated protein kinase (MAPK) and c-Jun N-terminal kinase (JNK) pathways participate in the 1-D-MT mediated upregulation of IDO1. IDO1 mRNA (left panel) and kyn release (right panel) of SKOV-3 cells treated with (**A**) 50 µM of the MEK1 inhibitor PD98059, (**B**) 20 µM of the p38 MAPK inhibitor SB203580 and (**C**) 20 µM of the JNK inhibitor SP600125 or control 1 h before addition of 1 mM 1-D-MT analyzed by qRT-PCR after 24 h and HPLC after 48 h, respectively. Experiments were performed in triplicate. Data are mean ± SEM. * (p<0.05).

We then explored signalling pathways involved in the upregulation of IDO1 in response to 1-D-MT treatment. IFN-mediated STAT1 phosphorylation is involved in the induction of IDO1 in many different cells and tissues [30], but knockdown of STAT1 by siRNA did not decrease the kyn production of 1-D-MT-treated cells (Fig. 7A). In line with this result, 1-D-MT did not induce the mRNA expression of IFN-β or IFN-γ (Fig. 7B). Mitogen activated protein kinase (MAPK) pathways have been reported to be modulated by the racemic mixture of 1-MT and thereby influence the polarization of dendritic cells (DC) [31]. We therefore tested whether inhibition of of MAPK signalling affected the 1-D-MT-mediated increase in IDO1 expression. Inhibition of ERK phosphorylation by PD98059 affected IDO1 mRNA expression and kyn release neither in untreated nor in 1-D-MT treated cells (Fig. 8A). While inhibition of p38-MAPK phosphorylation by SB203580 [32] slightly reduced IDO1 mRNA in untreated cells, it almost completely mitigated the increase in IDO1 mRNA expression in response to 1-D-MT (Fig. 8B). The slight inhibition of IDO1 transcipt in untreated cells did not translate into significantly reduced kyn release, while the reduction in kyn release became significant in 1-D-MT treated cells (Fig. 8B). Similar results were obtained when inhibiting JNK by SP600125 (Fig. 8C) [33]. Collectively, these data suggest, that p38 MAPK and JNK signalling are involved in mediating the induction of IDO1 in response to 1-D-MT.

## Discussion

In the past IDO inhibition was mostly achieved using the racemic mixture of 1-MT [34]. As it became apparent that IDO inhibition may be a promising target for cancer therapy, the individual stereoisomers of 1-MT were investigated in more detail [5], [35]. Although 1-L-MT was shown to more effectively inhibit IDO1 in enzyme assays and in cancer cell lines, 1-D-MT showed superior anti-tumor activity in mouse models and was therefore chosen for clinical trials [35]. A subsequent study suggested that the superior anti-tumor activity of 1-D-MT may result from inhibition of the IDO2 isoform [27]. Recent studies indicated that 1-D-MT inhibits IDO activity neither in dendritic cells nor in tumor cells [26], [28] and does not effectively restore IDO-induced arrest of T-cell proliferation [36]. In our study, 1-D-MT suppressed T cell proliferation when constitutively IDO1-expressing SKOV-3 cells were cocultured with mixed lymphocyte reactions (Fig. 2E,F). Investigation of the underlying mechanisms surprisingly revealed that 1-D-MT increased the kyn production of cancer cells with IDO1 activity (Fig. 4, 6) due to upregulation of IDO1 mRNA and protein expression (Fig. 5, 6). The upregulation of IDO1 expression and activity was observed only in cancer cells with either constitutive or IFN-γ-induced IDO1 expression (Fig. 5, 6). Upregulation of IDO1 by 1-D-MT in many cancer cells was most prominent at moderate concentrations of IFN-γ that are likely to resemble physiological concentrations (Fig. 6C). Low concentrations of IFN-γ may not have induced sufficient IDO1 expression (Fig. 6C), possibly explaining why 1-D-MT did not increase IDO1 at these concentrations, while stimulation with very high concentrations of IFN-γ may have resulted in maximal IDO1 induction (Fig. 6C), thus explaining why further increase by 1-D-MT was not observed. In all of the examined IDO1-expressing cells an increase in IDO1 expression and kyn release was observed in response to treatment with 1-D-MT, indicating a general mechanism involved in 1-D-MT-mediated IDO1 induction (Fig. 4, 5, 6).

In a previous study, the racemic mixture of 1-MT has been reported to modify the polarization of dendritic cells (DC) by modulating MAPK [31]. Inhibition of p38 MAPK phosphorylation prevented the increase in IDO1 mRNA and kyn production by 1-D-MT (Fig. 8B), suggesting that p38-MAPK participates in 1-D-MT mediated signalling. In line with this result, p38 MAPK has previously been shown to contribute to the induction of IDO1 in a leukaemia cell line and in DC [37], [38]. Also inhibition of JNK signalling mitigated the induction of IDO1 mRNA and kyn release in the presence of 1-D-MT (Fig. 8C). Inhibition of JNK phosphorylation has recently been described to decrease IDO1 expression induced by LPS in mouse microglia [39]. 1-D-MT mediated modulation of p38-MAPK and JNK suggests that 1-D-MT may have more effects than just the upregulation of IDO1.

To our knowledge this is the first report of 1-D-MT-mediated effects on gene expression in human cells. The stereoisomer of 1-D-MT, 1-L-MT was recently reported to suppress the IFN-γ-induced expression of IDO1 in mouse rectal carcinoma cells [40]. In addition, it has previously been described that 1-MT influences the maturation of DC independently of IDO [31]. However the racemic mixture of 1-MT was used in this study and it is therefore not known which stereoisomer was responsible for the observed effects [31]. It remains to be elucidated whether 1-D-MT exerts more effects on gene expression than the regulation of IDO1. In contrast to IDO1, the trp-catabolising enzyme IDO2 was not induced by 1-D-MT. This finding underlines the notion that IDO1 and IDO2 are differentially regulated on a transcriptional level [27]. Possible other effects of 1-D-MT on gene expression may contribute to the high anti-tumor efficacy of 1-D-MT observed in mouse tumor models [35]. Significant differences in IDO expression and regulation exist between humans and mice [29], [41], which could account for the observed discrepancies regarding 1-D-MT action in mouse models and human cells. In a study using 1-D-MT in SIV-infected rhesus macaques, a model more closely resembling humans than mouse models, Boasso and colleagues observed that kyn plasma levels were not reduced but rather induced during treatment with 1-D-MT [42]. Increased IDO1 mRNA expression in lymph nodes of macaques after 1-D-MT treatment was interpreted as a compensatory counterregulatory mechanism activated by 1-D-MT, which may have accounted for the lack of effect on plasma kyn [42]. Our data show that upregulation of IDO1 and kyn take place also in isolated human cells, in which a counterregulatory mechanism that mediates immunosuppression is unlikely.

As there is evidence that IDO1 may restrict tumor growth as a mediator of tumoricidal IFN-γ in experimental models and patients [16], [17], [18], [19], [20], [21], [22] and as IDO1 expression in tumors positively correlated with progression-free survival and long-term survival in some studies [23], [24] it is tempting to speculate that the induction of IDO1 by 1-D-MT may actually account for some of the anti-tumor effects of 1-D-MT.

In conclusion, we have identified the upregulation of IDO1 expression in human cancer cells as a profound effect of 1-D-MT, a compound currently used in clinical studies in patients with relapsed or refractory solid tumors with the aim of inhibiting (IDO)-mediated tumor immune escape. IDO1 expression is known to be immunosuppressive and may enhance tumor immune escape, but it has also been implicated in direct anti-tumor effects. More studies are needed to better understand the role of IDO in cancer biology and the potential use of 1-D-MT as an anti-cancer agent.

## Materials and Methods

### Cell culture and reagents

SKOV-3 and NIH:OVCAR-3 ovarian carcinoma cells were cultured in McCOY's 5A Medium (BioConcept, Allschwil, Switzerland) supplemented with L-trp as indicated (Sigma-Aldrich, Taufkirchen, Germany), 300 mg/L Glutamine (Carl Roth, Karlsruhe, Germany), 10% FBS (Thermo Fisher Scientific Inc., Waltham, MA, USA) and 100 U/mL penicillin and 100 µg/mL streptomycin (PAA Laboratories, Pasching, Austria). HeLa cervical carcinoma cells, A375 malignant melanoma cells, LN18, LNT229, T98G and U251 malignant glioma cells were maintained in Dulbecco's modified Eagle's medium (DMEM, PAA) containing 10% FBS (Thermo Fisher Scientific Inc) and 100 U/mL penicillin and 100 µg/mL streptomycin (PAA Laboratories). Peripheral blood mononuclear cells (PBMC) were isolated from five healthy, non-related blood-donors by density-gradient centrifugation using lymphocyte separation medium LSA 1077 (PAA Laboratories) and cultured in RPMI 1640 (PAA Laboratories) containing 10% FBS (Thermo Fisher Scientific Inc) and 100 U/mL penicillin and 100 µg/mL streptomycin (PAA Laboratories). All cells were routinely tested for contamination by the Multiplex cell Contamination Test [43]. Cultures were incubated at 37°C in a 5% CO_2_ atmosphere.

20 mM stock solutions of 1-methyl-D-tryptophan (1-D-MT, lot numbers: 09315BH, 08007EJ) and 1-methyl-L-tryptophan (1-L-MT, lot numbers: 08023HE, 15399MJ) (Sigma-Aldrich) were prepared by dissolving the inhibitors in 0.1 N NaOH. The pH was adjusted to 7.5 using hydrochloric acid. To avoid contamination of the cell cultures, the stock solutions were filfotered through 0.2 µm filters. IFN-γ was purchased from Immunotools (Friesoythe, Germany). ERK phosphorylation was inhibited using the MEK1 inhibitor PD98059 (Cell Signaling Technology, Beverly MA, USA). The c-Jun N-terminal kinase (JNK) inhibitor SP600125 and the inhibitor of p38 kinase phosphorylation SB203580 were purchased from Enzo Life Sciences (Lörrach, Germany).

### High performance liquid chromatography (HPLC)

High performance liquid chromatography (HPLC) analysis was performed according to [44] using a Beckman HPLC with photodiode array (PDA) detection and Lichrosorb RP-18 column (250 mm×4 mm ID, 5 µm, Merck, Darmstadt, Germany). Kyn release and trp degradation were measured in the medium of 3 * 10^5^ cells in 2 mL McCOY's 5A Medium (BioConcept) containing 10% FBS (Perbio), 100 U/mL penicillin and 100 µg/mL streptomycin (PAA Laboratories) supplemented with 0–125 µM L-trp (Sigma-Aldrich). The medium was harvested from 6 well plates at the indicated time points, centrifuged and frozen until further analysis. After thawing, the samples were supplemented with trichloroacetic acid for protein precipitation, centrifuged and 100 µl of the supernatant was analyzed by HPLC. Standard curves were generated with L-kyn and L-trp (Sigma-Aldrich) in the same medium. Since FBS contains kyn, low kyn concentrations (∼1 µM) were detected in all samples and medium without cells was always measured for comparison.

### Quantitative (q)RT-PCR

Total RNA was isolated with the Qiagen RNAeasy RNA isolation kit (Hilden, Germany) and DNA was synthesized with the Applied Biosystems reverse-transcription-Kit (Foster City, CA, USA) according to manufacturer's instructions. QRT-PCR was preformed in an ABI 7000 thermal cycler with SYBR Green PCR Mastermix (Applied Biosystems) according to standard protocols. PCR reactions were checked by including no-RT-controls, by omission of templates and by both melting curve and gel analysis. Standard curves were generated for each gene. Relative quantification of gene expression was determined by comparison of threshold values. All results were normalized to GAPDH, which varied neither with IFN-γ nor 1-D-MT treatment.

Primer sequences were (5′-3′ forward, reverse):

CTCTCTGCTCCTCCTGTTCGAC, TGAGCGATGTGGCTCGGCT (GAPDH),
TTCAGTGCTTTGACGTCCTG, TGGAGGAACTGAGCAGCAT (IDO1),
TGCTTCATGCCTTTGATGAG, GAAGGCCTTATGGGAAGGAG (IDO2),
ACTGCCTCAAGGACAGGATG; AGCCAGGAGGTTCTCAACAA (IFN-β),
TCGGTAACTGACTTGAATGTCCA; TCCTTTTTCGCTTCCCTGTTTT (IFN-γ),
AGGAAAAGCAAGCGTAATCTTCA; TATTCCCCGACTGAGCCTGAT (STAT1),
GGTTCCTCAGGCTATCACTACC; CAGTGTCGGGGAATCAGGT (TDO)

### siRNA experiments

To knockdown IDO1 (INDO) and STAT1 ON-TARGETplus SMART-pool siRNA by Dharmacon RNA Technologies (Lafayette, CO, USA) was used.

The sequences were as follows:

*Human INDO, NM_002164*, sense, 5′-UCACCAAAUCCACGAUCAUUU-3′, antisense, 5′-PUAUGCGAAGAACACUGAAAUU-3′; sense, 5′-UUUCAGUGUUCUUCGCAUAUU-3′, antisense, 5′-PUAUGCGAAGAACACUGAAAUU-3′; sense, 5′-GUAUGAAGGGUUCU GGGAAUU -3′, antisense, 5′-PUUCCCAGAACCCUUCAUACUU-3′; sense, 5′-GAA CGGGACACUUUGCUAAUU-3′, **antisense, 5′-PUUAGCAAAGUGUCCCGUUCUU-3′**
*Human STAT1, NM_139266*, sense, 5′-GCACGAUGGGCUCAGCUUUUU-3′, antisense, 5′-PAAAGCUGAGCCCAUCGUGCUU-3′; sense, 5′-CUACGAACAUGACCCUAUCUU-3′, antisense, 5′-PGAUAGGGUCAUGUUCGUAGUU-3′; sense, 5′-GAACCUGACUUCCAUG CGGUU-3′, antisense, 5′-PCCGCAUGGAAGUCAGGUUCUU-3′; sense, 5′-AGAAAGAG CUUGACAGUAAUU-3′, antisense, 5′-PUUACUGUCAAGCUCUUUCUUU-3′.

ON-TARGETplus siCONTROL Non-targeting Pool (D-001810-10-05, Dharmacon) and a transfection without siRNA were used as negative controls.

For transfection of siRNA, the Amaxa Nucleofector Kit V (Amaxa biosystems, Koeln, Germany) was used. Briefly, 3 * 10^5^ cells were resuspended in 100 µl of the nucleofector solution V and mixed with 1.5 µg of siRNA, then electroporated using program V005. Medium was changed after 24 h, analysis of the kyn content of the medium by HPLC, harvesting of the cells for RNA extraction or the generation of lysates and immunocytochemical analysis were performed after 48 h.

### Western blot analysis

Whole cell lysates were prepared in ice cold tris(hydroxymethyl)aminomethane hydrochloride (TRIS-HCl, 50 mM, pH 8,0; Carl Roth) containing 150 mM NaCl (J.T. Baker, Deventer, Netherlands), 1% Triton X-100 (AppliChem, Darmstadt, Germany), 10 mM EDTA (Gerbu Biotechnik, Gaiberg, Germany), 200 mM dithiothreitol (Carl Roth), 3% 2-mercaptoethanol (Sigma-Aldrich), 100 µM phenylmethylsulphonyl fluoride (PMSF), 10 µg/mL aprotinin and 5 µg/mL leupeptin (Carl Roth) and centrifuged at 4°C (10 min, 13 000 rpm). The protein concentration of the supernatants was determined using the Bio-Rad protein assay (Bio-Rad, Hercules, CA, USA) at 595 nm. The desired amount of protein (20 µg per lane) was separated by 10% SDS-PAGE and transferred to a 0.2 µm-pore nitrocellulose membrane (Whatman, Dassel, Germany). After 1 h of blocking in PBS supplemented with 0.2% Tween 20 (Sigma-Aldrich) and 5% bovine albumin fraction V powder (Carl Roth), the membrane was incubated with rabbit anti-IDO1 antibody (1∶2000, Alexis, Lausen, Switzerland) , rabbit anti-STAT3, mouse anti-phospho-STAT3 (Tyr705) (all 1∶1000, Cell Signaling, Danvers, MA, USA) or goat anti-GAPDH (1∶2000, Abcam, Cambridge, UK) as loading control, over night at 4°C. After a 2 h incubation at room temperature with secondary antibodies donkey anti-rabbit HRP conjugated (1∶5000, GE-Healthcare, Buckinghamshire, UK) or donkey anti-goat HRP conjugated (Santa Cruz Biotechnology), protein detection was performed using ECL Plus reagent (GE Healthcare).

### Immunocytochemistry

SKOV-3 cells were grown on poly-L-lysine (Sigma-Aldrich) covered glass slides in McCoy's 5A media (BioConcept) for 24 h. Cells were fixed with methanol (VWR, Darmstadt, Germany). Antigen-retrieval was performed with 0.25% Triton-X (AppliChem) in PBS (PAA Laboratories, Pasching, Austria). Unspecific binding was blocked with 5% BSA (Carl Roth) in PBS for 1 h. Cells were incubated overnight with rabbit anti-IDO1 antibody (1∶100, Alexis, Lausen, Switzerland) at 4°C. Secondary antibody donkey anti-rabbbit Cy3 (1∶50 JacksonImmuno, West Grove, USA) was applied for 45 min at room temperature. Stained slides were covered with DAPI-containing Vectashield Hardening Mounting Medium (Vector Laboratories, Burlingame, USA). Pictures were acquired using a BX51 microscope with cell∧ F software (Olympus, Hamburg, Germany)

### Mixed leukocyte reaction (MLR)

SKOV-3 cells were seeded in flat-bottom 96-well plates in McCoy's 5A medium (BioConcept) containing 10% FBS, 100 U/mL penicillin and 100 µg/mL streptomycin. 24 h after seeding SKOV-3 cells were pretreated with 1 mM 1-D-MT (Sigma-Aldrich). After another 32 h 2 * 10^5^ irradiated (30 Gy) PBMC as stimulators and 2 * 10^5^ PBMC from unrelated donors as responders were added. 1-D-MT and L-trp were added to the cultures at 1 mM (1-D-MT) or 250 µM (L-trp) immediately after addition of the PBMC. 6-day MLR were performed and cultures were pulsed with [3H]-methylthymidine (Perkin Elmer, Wellesley, MA, USA) for the last 18 h. The cells were then harvested, and radionuclide uptake was measured by scintillation counting. SKOV-3 wells with a knockdown of IDO1 and SKOV-3 control cells that were transfected with non-targeting siRNA were seeded in flat-bottom 96 wells plates 24 h after transfection. After 8 h 2 * 10^5^ irradiated (30 Gy) PBMC as stimulators and 2 * 10^5^ PBMC from unrelated donors as responders were added. After 72 h cultures were pulsed with [3H]-methylthymidine for the last 18 h. The cells were then harvested, and radionuclide uptake was measured by scintillation counting.

### Cell cycle analysis

Cells were incubated with 10 µM BrdU for 1 h, harvested and then fixed with 70% (v/v) methanol (VWR). To uncover the DNA, cells were treated with a PBS – based buffer (PAA) containing 0.1 M hydrochloric acid (VWR) and 0.3% Triton X-100 (AppliChem) and boiled in water. Finally, cells were incubated with Alexa-Fluor 647 mouse anti BrdU antibody (clone 3D4, BD Biosciences, Heidelberg, Germany). 20 µg/mL DAPI (Sigma-Aldrich) was added 1 minute before analysis by flow cytometry (FACSCantoII, BD Biosciences).

### Measurement of T cell proliferation in MLR/SKOV-3 cocultures by CFSE staining and flow cytometry

2000 SKOV-3 cells were seeded in flat-bottom 96-well plates in McCoy's 5A medium (BioConcept) containing 10% FBS, 100 U/mL penicillin and 100 µg/mL streptomycin 24 h before PBMC isolation. Irradiated (30 Gy) PBMC as stimulators and PBMC from unrelated donors as responders at 2×10^6^/ml in RPMI 1640 containing 10% FCS, 100 U/mL penicillin and 100 µg/mL streptomycin were stained with 5 µM CFSE (5-(and 6)-Carboxyfluorescein diacetate, succinimidyl ester, Molecular Probes, Eugene, OR, USA) at 37°C for 5 min. The cells were then washed twice with culture medium and the stained cells (4×10^5^/well, 200 µl) were seeded on top of the SKOV-3 cells and stimulated with indicated concentrations of 1-D-MT. After 6 days of coincubation, the proliferation of the CFSE-stained lymphocytes was analyzed by flow cytometry (FACSCantoII, BD Biosciences). Unstained cells were included in all experiments and were used for normalization. Proliferation was analysed using FlowJo Flow Cytometry Analysis Software (Treestar Inc. Ashland, OR, USA).

### Statistical analysis

Data are expressed as mean ± SEM. Experiments were repeated at least three times with similar results. Analysis of significance was performed using the Student's t-test (SigmaPlot, Systat Software Inc., San Jose, CA, USA). P values<0.05 were considered significant *.
